# Constructing a Chinese electronic medical record corpus for named entity recognition on resident admit notes

**DOI:** 10.1186/s12911-019-0759-2

**Published:** 2019-04-09

**Authors:** Yan Gao, Lei Gu, Yefeng Wang, Yandong Wang, Feng Yang

**Affiliations:** 1 School of information Science and Engineering, Central South University, Changsha, People’s Republic of China; 2 Creator Information PTY LTD, Changsha, People’s Republic of China; 3 School of software, Central South University, Changsha, People’s Republic of China

**Keywords:** Resident admit notes, Named entity extraction, Annotation scheme, Deep neural network

## Abstract

**Background:**

Electronic Medical Records(EMRs) contain much medical information about patients. Medical named entity extracting from EMRs can provide value information to support doctors’ decision making. The research on information extraction of Chinese Electronic Medical Records is still behind that has done in English.

**Methods:**

This paper proposed a practical annotation scheme for medical entity extraction on Resident Admit Notes (RANs), and a model which can automatic extract medical entity. Nine types of clinical entities, four types of clinical relationships were defined in our annotation scheme. An end-to-end deep neural network with convolution neural network and long-short term memory units was applied in our model for the medical named entity recognition(NER).

**Result:**

We annotated RANs in three rounds. The overall F-score of annotation consistency was up to 97.73%. And our NER model on the 255 annotated RANs achieved the best F-score of 91.08%.

**Conclusion:**

The annotation scheme and the model for NER in this paper are effective to extract medical named entity from RANs and provide the basis for fully excavating the patient’s information.

## Background

Electronic medical records (EMRs) contain rich information, such as text, symbols, charts, graphs, figures, images and other digital information generated by the medical personnel during the medical process. With the continuous development of information technology, it has led to an unprecedented expansion of the electronic medical data in China. At the same time, research has becoming more and more focused on exploiting the value of the medical information. However much of unstructured medical information in EMR is not directly usable by existing clinical applications. Named Entity Recognition is a technology of Natural Language Processing(NLP) that can extract the medical named entities or clinical concepts in EMR to discover the knowledge embedding in the clinical reports. This is the main task focused in our research.

There is much research work have been done about clinical annotation corpus, both in English and Chinese [1]. In 2006, Meystre et al. [2] constructed a type of annotated corpus for 80 common medical problems (concept or entity), and Robert et al. [3] randomly selected 50 clinical records, x-ray and pathological reports, annotated entity, modification, entity relationship and temporal information, extended the entity type to six categories for the first time. In 2009, south et al. used electronic clinical records of 316 patients with inflammatory bowel disease [4] (including signs and symptoms, diagnosis, procedure, drugs). In their annotation work, the attribute and description context feature were determined, the study of modification (assertion) was discussed in detail. The marking process was completed by two annotators, KAPPA-value and the concept evaluation selected F-value were used to measure the consistency of annotation work. Uzuner et al. [5], in 2010 i2b2 [6, 7] published annotation concept, assertion and relationship corpus, based on the definition of semantic type of UMLS [8], the concept was divided into three categories: medical problem, test and treatment, three kinds of relations between the concepts are identified, the type of modification of medical problems was also carefully divided into six categories. This work was completed by medical experts and F-value was used for consistency evaluation. In 2012, the task of the i2b2 [9] challenging was to extract temporal information in clinical texts. In more detail, it was to infer and query the timeline of each patient’s clinical events related to three types of information: events, time expressions and their time relationships.

Comparing with the work of annotation on English data, analogous work of Chinese Electronic medical records (CMER) starts late. Referring to the classification system in the i2b2 concept annotation guidelines in 2010, Lei et al. [10] used the electronic medical records of Peking Union Medical College hospital in 2013 and constructed a corpus of 800 documents, including 400 admission summaries and 400 discharge summaries. They subdivided treatment into drugs (medication) and procedure. The annotation work was completed by two doctors, and tagged 40 documents for consistency evaluation. Word segmentation has a great influence on the effect of Chinese information extraction, in order to train the joint model of word segmentation and named entity recognition. Xu et al. [11] constructed a corpus of 336 discharge abstracts (DA) in 2014. The entity classification in Xu’s paper and that in Lei’s were similar, but anatomy was considered by the former. In 2016, the team of Harbin institute of technology developed a corpus [12]. They used multiple de-identified medical records from the affiliated hospital of Harbin Medical University and selected the first course of disease and DA, which covered all the 35 departments in the hospital. The entities were classified into four categories: inspection, symptom, disease and treatment, and the entity relationship had six categories. The consistency was evaluated by using F-value.

In the existing annotation schema on CEMR, the comprehensive research on symptoms is less. But in the medical field, symptom is useful in exploring the causes, development and evolution of the patient’s morbidity. Resident Admit Notes (RANs) contain rich information of symptom. Based on Chinese RANs, we defined our annotation scheme, including nine types of clinical entities and four types of clinical relationships around symptoms. Comparing with previous work, “body part” entity and “temporal word” entity are added in our annotation work, and “inspection” entity and “laboratory test” entity is distinguished. Then we constructed an improved and optimized four-layer deep learning framework which was applied to extract maned entity in RANs automatically and achieved good results. In this way, we have successfully built the annotation corpus for medical named Entity Recognition based on Chinese RANs.

## Methods

### Annotation Schema

**Fig. 1:**
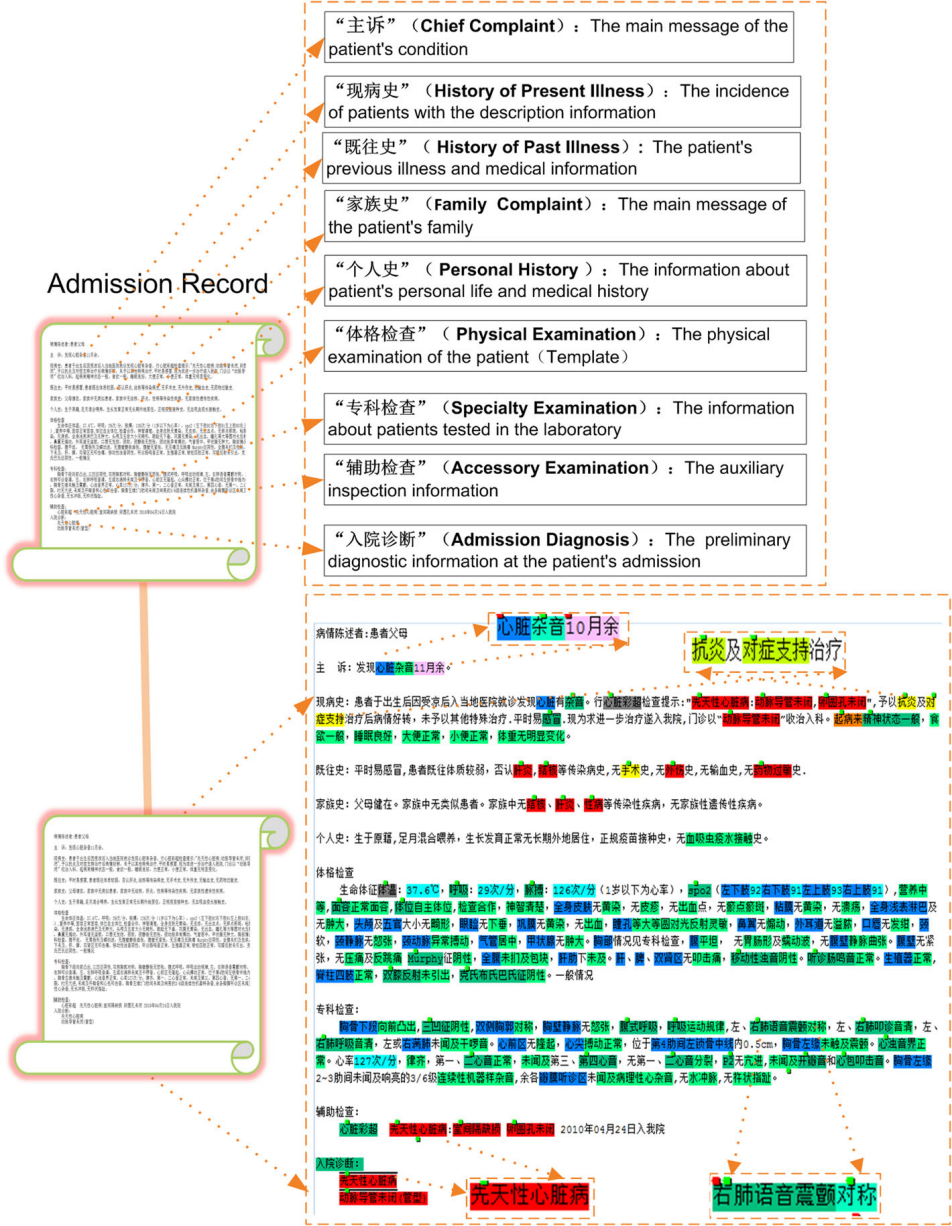
An example of admission record with annotated entities

This study uses 255 authentic admission records from a famous hospital in Hunan province, China. The RANs come from a variety of departments in the hospital. We present an example of the admission record with the annotated entities in Fig. 1. It is notable that the contents of RANs contain abundant information: structural features such as section headings are clearly identifiable; the main content contains useful medical information including patients’ chief complaints, present history, past history, family history, physical examination, laboratory test, drugs and admission diagnosis. The annotated entities and their types are also illustrated in the right side of the figure, and some examples are described as below:

“心脏杂音10月余”[heart murmur for more than 10 months], 心脏(“heart”) = “Body part”(blue); 杂音(“murmur”) = “Medical discovery”(light green); 10月余(“more than 10 months”) = “Temporal word”(pink);

“抗炎及对症支持治疗”[anti-inflammatory and symptomatic supported treatment], 抗炎(“anti-inflammatory”) and 对症支持(“symptomatic supported”) = “Treatment”(yellow); “先天性心脏病”[congenital heart disease], this whole is marked as “Disease”;

“右肺语音震颤对称”[speech tremor in the right lung is symmetrical], 右肺语音震颤(“speech tremor in right lung”) = “Inspection”(green), 对称(“symmetrical”) = “Medical discovery”(light green).

Relationships are not presented in the diagram, but we will give detailed explanations in later sections.

#### Annotation principal

Based on the i2b2 annotation guidelines, we developed our annotation guidelines. And after repeated discussions, we formulate the following principles which will be strictly followed:

- Punctuation should not be annotated separately, and is not included in the annotation as far as possible. This is to minimize the interference of symbols on the meaning of annotated entities. The punctuations will be annotated if they have special meanings.
- Entity annotation is not overlapped or nested. We will not annotate the same object as different entities.
- For some complex or ambiguous situations, we may make some special appointments based on the annotation types and doctors’ suggestions, similar objects will be annotated according to these appointments.
- The negation words won’t be separately annotated unless it is inseparable from the entity.

#### Medical entity

Admission records are the first-hand material for researching the condition of the patients during their hospitalization. They contain important information for analyzing the patient’s inchoate symptom and disease. Based on the patient’s symptoms, we have formulated nine types of entities: Medical discovery, Body part, Temporal word, Disease, Medication, Treatment, Inspection, Laboratory test and Measurement. Except “Measurement”, all entities are analysed in details in this section. The illustrating examples are expressed by “equation”. The left part of “equation” is original texts from RANs, the left part of “equation” is the entity.

##### Medical discovery

This type of entity is annotated in light green and we call it “Discovery” in short. It is composed of three types of clinical finding: patient’s normal condition, normal results obtained from inspection or test and the symptoms of patient. The normal discoveries are meaningful for analyzing different symptoms or different manifestations. The abnormal conditions include abnormal conditions, abnormality findings in inspection or test. We call them “Symptom”. In the entity relationship analysis below, we only focus on the relationships of entities about symptom. All the following examples are based on word-level Chinese to English translation (grammar isn’t considered completely).

“Normal condition” refers to the normal descriptions of the patient’s current condition by inspection or examination. In admission records, many of them follow a fixed pattern, but the results vary from patient to patient. Several typical examples are described in the following:

- 自起病以来食欲一般[since symptoms produce, appetite normal] = 食欲一般(“appetite normal”).
- 无血吸虫疫水接触史[no schistosome contaminated water contacting history] = 血吸虫疫水接触(“schistosome contaminated water contacting”).

“Inspection and laboratory test (I&Lt) normal” refers to the normal results obtained by inspection or laboratory test of a patient.

- 移动性浊音阴性[mobile dullness negative] = 阴性(“negative”).
- 肝脾未触及[Liver and spleen untouched] = 触及(“touched”).
- 肝脾未触及肿大[liver and spleen untouched swelling] = 肿大(“swelling”).

There are two notable points here are notable: 1) “mobile dullness” belongs to the inspection method, so we classify it as “inspection”; 2) “未触及”(“untouched”),“未听见” (“unheard”) and so on are not only the negation words, but they have specific clinical meanings. The sentence “liver and spleen untouched” means the liver and spleen are not swollen. So “touched” is identified as “Discovery” (the affirmative meaning is marked only). In example “liver spleen untouched swelling”, the clinical object “swelling” is modified by “untouched”, so “untouched” is identified as a negative word according to the principle 4.

##### Symptom

This entity refers to abnormal descriptions of the patient which is known or checked for the condition. Several typical examples are described in the followings:

- 疼痛时伴有右下肢活动受限[pain accompanied by limited activity of right lower limb] = (疼痛(“pain”), 活动受限(“limited activity”)).
- 稍感胸闷气短[mild chest tightness and shortness of breath] = (胸闷(“chest tightness”), 气短(“shortness of breath”)).

##### Body part

This entity is marked in blue. We consider “Body part” as a distinct feature that can be used to inform the doctor the location of symptom in practice. We will not include the orientation information at the time of marking, for example:

- 心脏, 胸腔无杂音[no noise in heart] = (心脏(“heart”), 胸腔(“thorax”).
- 双肺叩诊音清[both lung percussion sound clear] = (“”).

If the entity “Body part” is contained in “Disease”, “Medication”, “Inspection”, “ Laboratory test” or “Treatment”, we will mark the whole as “Disease”, “Medication” etc.

##### Temporal word

This entity is marked in pink. The temporal words related with symptom will be benefit to the further research of the cause, evolution and diagnosis of symptom. This special feature has been studied in the 2012 i2b2 challenge. The temporal words only related with symptom are regarded as a kind of entity in our work.

- 发病十月余[symptoms persists for ten months] = 十月余(“ten months”).
- 晚饭后突发脑溢血[sudden cerebral haemorrhage after dinner] = 晚饭后(“after dinner”).

The temporal words we need to mark are the Chinese words related to disease or symptom, and are not the exact dates or time. These temporal words or phrases can reflect the cause, attribute or development process of the symptom to a certain extent.

#### Disease

This type of entity is marked in red, which is important and complex. There are many studies aimed at it in both English and Chinese papers. In our work, we only focus on the extraction of the entity “Disease”, not its complex causal relationship. There are two reasons: 1.the disease process of production, treatment and cured are extremely complex and changeable, RANs cannot well represent them; 2.“Disease” is the conclusion of symptoms after overall diagnosis.

- 无药物过敏史[no drug allergy history] = 药物过敏(“drug allergy”).
- 入院诊断:先天性心脏病, 动脉导管未闭(管型)[admission diagnosis: congenital heart disease, patent ductus arteriosus(tube)] = (先天性心脏病(“congenital heart disease”), 动脉导管未闭(管型)“patent ductus arteriosus(tube)”).

##### Medication

This entity is marked in purple. This entity mainly consists of three aspects: medicine history, medication in doctor’s order and methods of drug therapy.

- 予以捷甘清, 亮菌甲素护肝退黄治疗后[after using JieGanqing, bright bacteria A for liver and jaundice treatment] = (捷甘清(“JieGanqing”), 亮菌甲素(“bright bacteria A”)).
- 曾多次服用感冒药和处方药[have taken cold medicine and prescription drugs many times] = (感冒药(“cold medicine”), 处方药(“prescription drugs”)).

The specific drugs or unspecific brand of drugs are both regarded as “Medication”.

##### Treatments

This entity marked in yellow includes surgical treatment and non-surgical treatment, drugs in treatment will be classified into “medication”. We treat both treatment process and therapeutic purpose as “Treatment”.

- 无手术史, 无外伤史, 无输血史[no operation history, no trauma history, no blood transfusion history] = (手术(“operation”), 外伤(“blood transfusion”)).
- 予以抗炎及胰岛素注射治疗后病情好转[after using anti-inflammatory and Insulin injecting treatment, the condition improve] = (抗炎(“anti-inflammatory”), 注射治疗(“injecting treatment”)).

##### Laboratory test

This entity marked by dark green indicates checking a sample of the blood, urine, or body tissues, a specialist or a doctor analyzes the test sample to see if the results fall into the normal range. The typical example is:

- 肝功能ALT537U/L[liver function] = (肝功能(“liver function”), ALT).
- 在急诊查随机血糖24.6 mmol/L[randomly checking blood sugar in emergency room] = (随机血糖“random blood sugar”).

We noted that both parameters and processes in laboratory tests as “Laboratory test”, which is also applicable similarly in the entity “Inspection”.

##### Inspection

This entity marked by gray is defined as the medical treatment that does not require the extraction of body samples to obtain the physical condition of the patient.

- 心脏彩超示[echocardiography display] = 心脏彩超(“echocardiography”).
- 生理反射存在, 巴氏征阴性[physiological reflex exists, Papanicolao negative] = (生理反射(“physiological reflex”), 巴氏征(“Papanicolaou”)).

#### Relationship

Based on the nature of the required information for doctors and the content of admission records, we derived the four relationships around symptom for medical decisions.

“*BrSy*”*:* The relationship between “Body part” and “Symptom” indicates the finding site of a symptom. The site involved may be more than one, and the same place may have different symptoms. “Body part” is mentioned in Lei’s research work, but he did not discuss the relationship between this entity and symptom in detail.

“*TrSy*”*:* The relationship between “Temporal word” and “Symptom” reflects the duration and point of the symptom, which is crucial for the study of the cause and evolution of the symptom.

*“IrSy” and “LrSy”:*“IrSy” and “LrSy” refer to the patient’s routine inspection and qualitative testing.

**Fig. 2:**
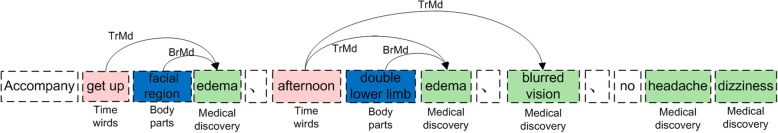
The relationship annotation of example1

**Fig. 3:**
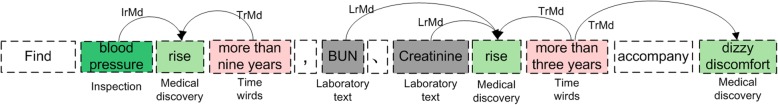
The relationship annotation of example2

**Fig. 4:**
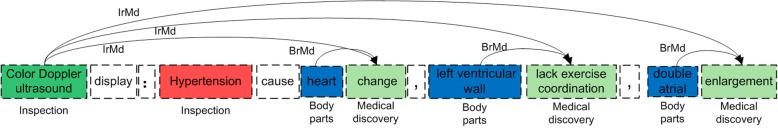
The relationship annotation of example3

Some examples are given below. These examples are translated from Chinese into English by word (grammar isn’t considered completely). The annotation details of these examples are described in Figs. 2, 3 and 4. The colors, arrows and texts in schematics are intuitive and meaningful.

- Example1:“晨起颜面部浮肿、下午双下肢浮肿、视力模糊、无头痛头晕”
- Example2:“发现血压升高9年余, 血尿素氮、肌酐升高3年余伴头晕不适”
- Example3:“彩超示:高血压所致心脏改变、左室壁运功欠协调、双房增大”

### Named entity recognition model

**Fig. 5:**
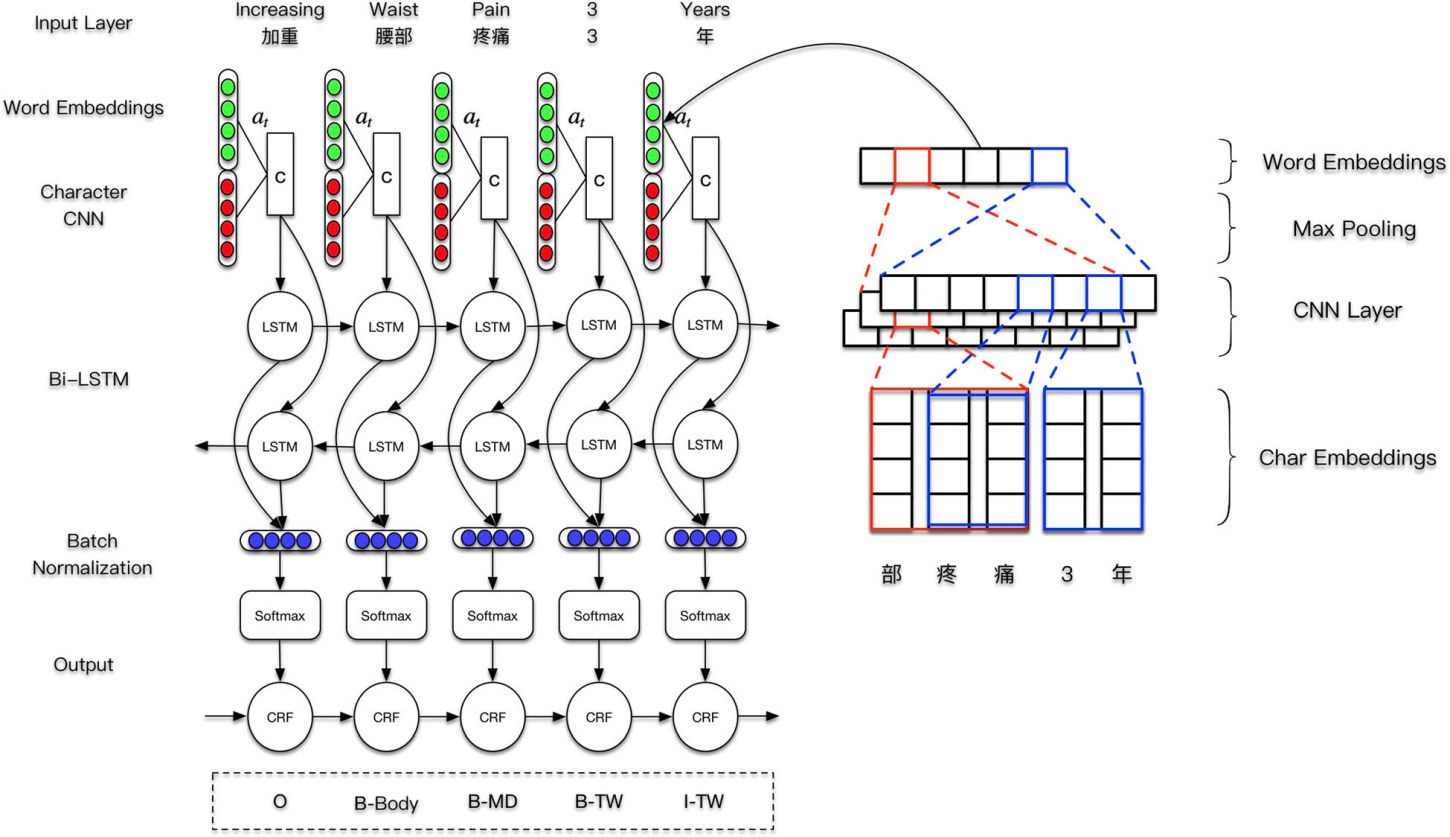
Architecture of the neural network

Our model for the named entity recognition tasks on our annotated corpus as well as some experiment results is described in this section. We build an end-to-end deep neural network model for the task. No handcrafted features are used, hence all features are learnt by the neural network. Figure 5 illustrates our neural network architecture, it consists of several layers, which are explained in the following sections.

#### Data representation

We formulate the NER task as a sequence classification task. Hence we employ the BIO tagging method where “O” means that word is not in an NE, “B” means the beginning of an NE, “I” means a part of an NE but not at the beginning. The sequence classification task is done at the sentence level. We split the corpus into sentences using new line and regular expressions. For word level NER we tokenize the sentence using jieba Chinese word segmentation tool. Each word is then split into characters. Words and characters are convert into feature indexes. We group the sentences into batches and padded the sentence using a special PAD token to make sentences in each batch the same length. The character in each word are also padded at the end to make sure every word has the same number of characters.

#### Word embedding

Word embedding maps word into a vector representation where semantically related word has similar vector representations. Each word in the input sentence is transformed into word embedding through an embedding lookup. It has been widely used in many NLP tasks, such as the Word2vec [13–15]. We trained our own word embedding using CWE [16], a Chinese embedding which can jointly learn word embedding and character embedding. We trained the embedding on the combination of three different corpus: The Chinese Daily Corpus, Chinese Wikipedia and 500 de-identified clinical notes taken from the hospital’s information system.

#### CNN for character representation

Convolution Neural Network(CNN) had been used in many English sequence annotation tasks [17, 18] to extract character embedding. It has shown that CNN is likely to extract morphological features such as prefix and suffixes effectively. For Chinese word, it composed by several characters and each character carry some meaning, which may compose the meaning of the word. Hence we hypothesis that CNN would be able to capture the importance of the character in each word. For each word, we employed a convolution and a max pooling layer to extract character-level representations. We use kernel size of 2, 3, 4 characters to convolve through the character sequences respectively. The three embeddings are concatenated together to form a new character embedding vector, and are then concatenated with the word embedding vector to form the input to the next level of the neural network.

#### Weighted combination of embedding

Another way to combine the character embedding with the word embedding is to use a weighted combination of the character embedding and word embedding. This mechanism is widely used in the attention networks [19]. The weights are predicted by a two layered network as following:

4 $$ z= sigmoid\left(U\bullet \tanh \left( Wx+ Vc+b\right)\ \right),x\_ out=z\bullet x+\left(1-z\right)\bullet c $$

Where *U*, *V*, *W* are weights learnt from the data, *x* is the word embedding vector, *c* is the character embedding, and *b* is the bias vector.

#### Bi-directional LSTM

Recurrent neural network (RNN) is a powerful neural model for sequence annotation tasks. It takes the input as a sequence of vector and returns another sequence. RNN is able to capturing long-distance dependencies, however, it suffers from the gradient vanishing problem [20]. We employed a bi-directional RNN with long short-term memory(LSTM) unit to encoding previous layers output into a unary named entity tag score as in other existing work [21]. It has shown that LSTM can effectively deal with variable length sentences and to resolve the gradient vanishing problem.

The word and character features are fed into a forward LSTM network and a backward LSTM network. The output of the forward and backward LSTM cells at each time step are concatenated together and then fed into a linear layer and a softmax layer to form a probability score for the entity tag. The softmax calculates a normalised probability distribution over all the possible label for a word.

#### CRFs

We used Conditional Random Fields (CRFs) as the output layer for the neural networks suggested in many work [21, 22]. CRFs condition the prediction on the previously predicted labels. In addition to the log-probability score for each tag, transition probabilities between different tags are learnt, and the Viterbi algorithm is used to find the most probable sequnce.

## Results

### Results of the annotation work

**Fig. 6:**
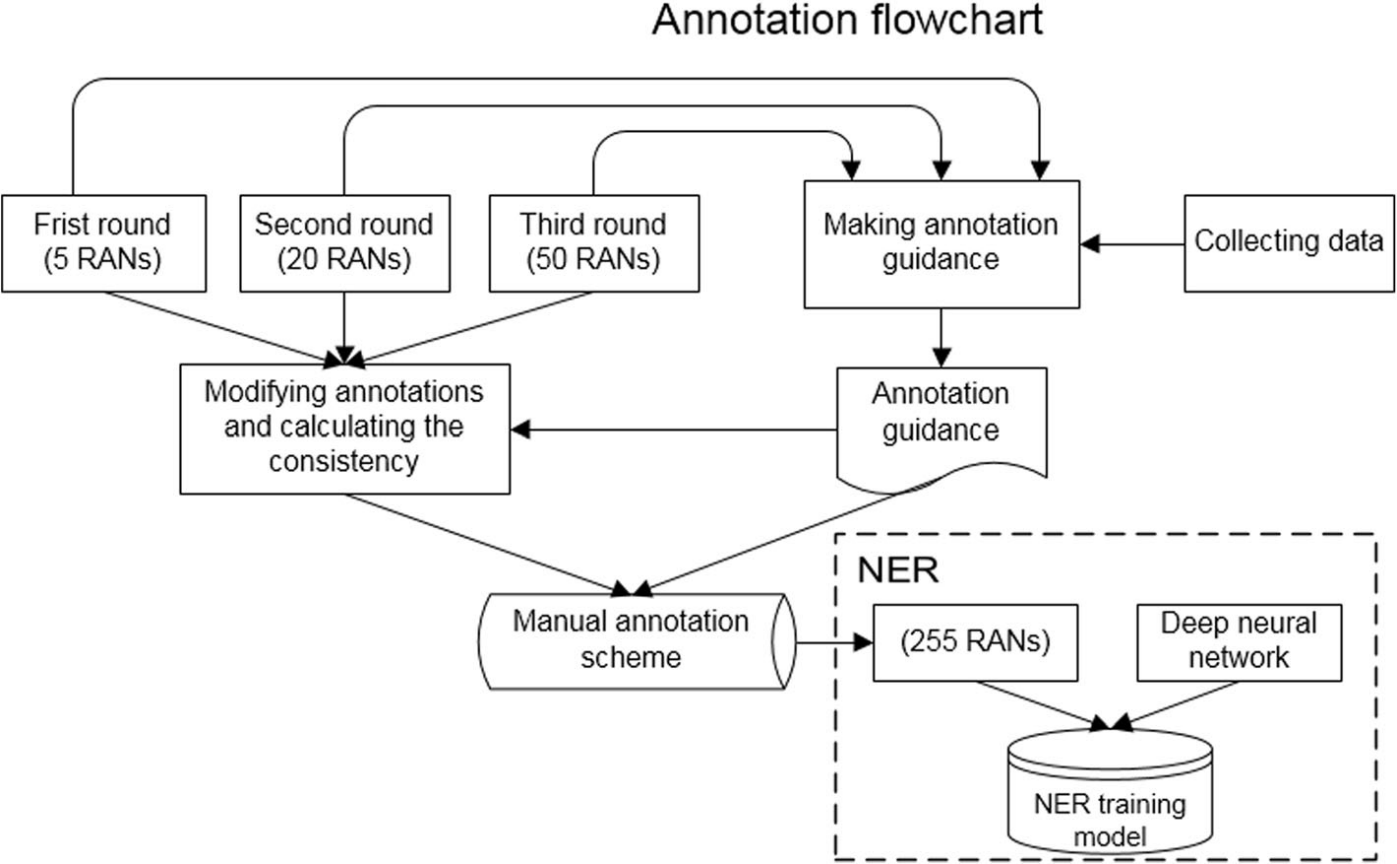
The illustration of the annotation process

We annotated 5/20/50 RANs in three rounds. The annotation process is shown in Fig. 6. The annotators made some discussions or consulted the medical professional during the annotation, but we keep the annotation work as independent as possible.

Three annotators (Annotator A, Annotator B and a manager) participated in this annotation work. The manager did statistical work. Everyone counted the number of annotations produced, and calculated the accuracy. The work was performed independently, and each person checked the annotation results for multiple times. The specific method is: to regard the results of a annotation as the standard answer, calculate the accuracy(P) and recall rate(R) of the results of consistent number, and then calculate the F value. Our statistical formulas are:

1 $$ P=\frac{\mathrm{The}\ \mathrm{total}\ \mathrm{number}\ \mathrm{of}\ \mathrm{result}\mathrm{s}\ \mathrm{consistent}\ \mathrm{with}\ \mathrm{A}\ \mathrm{and}\ \mathrm{B}}{\mathrm{The}\ \mathrm{total}\ \mathrm{result}\ \mathrm{number}\ \mathrm{of}\ \mathrm{B}} $$

2 $$ R=\frac{\mathrm{The}\ \mathrm{total}\ \mathrm{number}\ \mathrm{of}\ \mathrm{result}\mathrm{s}\ \mathrm{consistent}\ \mathrm{with}\ \mathrm{A}\ \mathrm{and}\ \mathrm{B}}{\mathrm{The}\ \mathrm{total}\ \mathrm{result}\ \mathrm{number}\ \mathrm{of}\ \mathrm{A}} $$

3 $$ R=\frac{P\times R\times 2}{P+R} $$

From Table 1, the average accuracy, racall, F1-score of annotation consistency in the first round was 77.11, 78.54, 77.82%. From Table 2, the average accuracy, racall, f1-score of annotation consistency in the second round was 90.25%. From Table 3, the average f1-score of annotation consistency in the end round was up to 97.73%. We can find that the annotation consistency is improved in three rounds. In the last round, only the f1-score of “Laboratory test” and “Laboratory test” are below 94%. “Laboratory test” has the lowest f1-score: 89.24%. The f1-score of other entities is above 94%. The f1-score of “Body part” is up to 99.11%. It can demonstrate our annotation schema is effective to guide people to make annotation on RANs.

**Table 1:** Entity annotation consistency statistic table on 5 RANs in the first round

	Annotate A	Annotate B	Consistent number	P/R/F(%)
Medical discovery	497	514	385	74.90/77.46/76.15
Temporal word	14	13	10	76.92/71.43/74.03
Inspection	102	110	70	63.63/68.63/66.04
Laboratory test	17	20	10	50.00/58.82/54.08
Treatment	29	26	22	84.62/75.86/80.00
Disease	85	93	77	82.80/90.59/86.52
Medication	3	2	2	100/66.67/80.00
Body part	288	276	238	85.51/81.94/83.69
Total(average)	1035	1053	812	77.11/78.54/77.82

**Table 2:** Entity annotation consistency statistic table on20 RANs in the second round

	Annotate A	Annotate B	Consistent number	P/R/F(%)
Medical discovery	2208	2175	1935	88.97/87.18/88.07
Temporal word	65	63	56	88.89/86.15/87.50
Inspection	510	465	426	91.61/83.53/87.38
Laboratory test	93	103	82	79.61/88.17/83.61
Treatment	97	102	94	92.17/96.91/94.48
Disease	384	382	342	89.53/93.97/97.70
Medication	11	11	11	100.00/100.00/100.00
Body part	982	989	942	92.25/95.93/95.59
Total(average)	4326	4200	3888	90.63/89.88/90.25

**Table 3:** Entity annotation consistency statistic table on50 RANs in the third round

Stage 3 (50DOCs)	Annotate A	Annotate B	Consistent number	P/R/F(%)
Medical discovery	5478	5488	5465	97.76/97.94/97.85
Temporal word	192	186	187	90.86/88.02/89.42
Inspection	1255	1247	1250	98.16/97.53/97.84
Laboratory test	308	315	310	87.94/90.58/89.24
Treatment	286	294	294	92.86/95.45/94.14
Disease	1061	1058	1065	99.05/98.77/98.91
Medication	35	34	34	97.06/94.29/95.65
Body part	2486	2472	2458	99.43/99.79/99.11
Total(average)	11,101	11,092	11,064	97.78/97.69/97.73

### Results of named entity recognition model

#### Dataset

The data used in the experiment were the annotated corpus described in the previous sections. Only named entities from corpus are used, resulting in 255 annotated document and 9 entity categories (including Measurement). The following table shows some statistics about the data we used in our experiment. Table 4 shows the statistics about the sentences, words and characters in the corpus, and Table 5 shows the distribution of entities in the corpus.

**Table 4:** Statistics of the data used in our experiment

	Sentences	Words	Features	Entities
Total	13,926	259,074	420,903	66,943
Average number of texts	54.61	1015.98	1650.6	262.52

**Table 5:** Distribution of entity types in the corpus

	Count	Percentage
Medical discovery	29,247	43.96%
Temporal word	1631	2.44%
Inspection	6915	10.33%
Laboratory test	2127	3.18%
Treatment	2601	3.88%
Measurement	2839	4.24%
Disease	5286	7.90%
Medication	1344	2.01%
Body part	14,953	22.34%
Total	66,943	100%

We employed 10-fold cross validation during training. The final results are the micro-average result of all folds.

#### Embedding initialization

We tried different embeddings sizes, for each experiment, we initialize the word embeddings using our trained word embeddings from CWE, described previously. For words that do not exist in the pre-trained embeddings, we use a vector of random value sampled from $\left[-\sqrt{\frac{3}{\mathit{\dim}}},+\sqrt{\frac{3}{\mathit{\dim}}}\right]$ where dim is the dimension of embeddings.

We use 300-dimensional character embeddings for representing each character. The embeddings are initialized using the character embedding output from CWE while we train the word embeddings. Unseen words are initialized using uniform samples from [− √ (3/*dim*), + √ (3/*dim*)] similarly.

We enabled fine-tune on both word and character embeddings when performing gradient updates during training.

#### Parameter tuning

We implement our neural network NER using Tensorflow framework. Learning is done by mini-batch stochastic gradient descent (SGD) where back-propagation is performed using Adadelta [23]. Different batch sizes are tested, where size of 20 yield the best performance. Moreover, we follow Pascanu et al. [24], and use a gradient clipping of 5.0, in order to reduce gradient exploding. To avoid overfitting, we apply batch normalization [25] and dropout [26] on hidden units in all layers in our models. Dropout rate is set to 0.5. We also use early stopping [27] based on the performance achieved on the development sets.

#### Experiment results

Table 6 presents our experiment results using the best parameter configuration and Table 7 presents the contribution of different hyper-parameters. The evaluation was using the standard Conll-2003 sared task evaluation method.

**Table 6:** Detailed named entity recognition performance

	Precision	Recall	F
Medical discovery	96.36	93.33	93.35
Temporal word	82.54	82.54	82.54
Inspection	90.97	91.66	91.31
Laboratory test	78.74	83.59	81.09
Treatment	89.29	82.51	82.4
Measurement	89.12	92.15	90.61
Disease	83.76	86.09	84.91
Medication	72.05	70.94	71.49
Body part	94.26	94.68	94.47
Total	90.76	91.4	91.08

**Table 7:** Performance of removing different techniques

	Precision	Recall	F
Best	90.76	91.4	91.08
No Dropout	89.85	290.12	89.98
No Attention	90.37	91.15	90.76
No Char Embeddings	84.61	84.81	84.71
LSTM for Char Embeddings	90.64	91.38	91.01

Without dropout, F-score of the NER model is 89.98%. Without attention mechanism, F-score of the NER model is 90.76%. Without Char Embeddings, F-score of the NER model is 84.71%. If we use LSTM for Char Embeddings, F-score of the NER model is 91.01%. Comparing with other different hyper-parameters, our NER model with attention mechanism and Char embedding has the best F-score: 91.08%.

## Discussion

### Discussion of annotation result

The overall F-score of annotation consistency indicates our schema’s effective. But there is still some annotation inconsistency. We analyse the inconsistency in the annotation results.

The F-score of “Medical discovery” is relatively high. Because “Medical discovery” has a wide definition, and the number is also the largest among all annotation types, a small number of annotation inconsistencies in “Medical discovery” won’t have a passive impact on it’s accuracy. There are two main reasons for the discrepancies: 1. Missing some discoveries, such as “自起病以来, 神志一般, 饮食正常”[since morbidity, conscious general, diet normal], 神志一般(“conscious general”) and 饮食正常(“diet normal”) are both belong to “Discovery”, but not annotated; 2.Confused with diseases, such as “颈静脉张” [jugular varices], (曲)张(“varices”) is a discovery, however, “外伤史” [history of trauma], 外伤(“trauma”) is “Disease”.

The F-score of “Laboratory test” has the lowest value: 89.24%. The reason is that “Laboratory” is in the confusion with “Inspection”. The annotations did not consult the information and ask the doctor carefully.

The number of “Temporal word” and “Medication” are small, hence a small number of inconsistencies in two annotator’s works will cause larger fluctuation in their F-score. The inconsistency of “Temporal word” is caused by the omissions and the wrong mark. “Medication” is clear in character, but small in quantity in RANs.

When annotating the entity “Treatment”, annotators may occasionally omit some conservative treatments or support treatments which are both the ineffective treatment. It is easy to form inertial thinking when annotating the entity “Body part”. When seeing a part, annotators mark it immediately without analysis of the context, which will cause the whole information of “Disease”, “Inspection” or other entity is stripped out. From the early lower statistics results of “Disease”, the two annotators, despite having a disease dictionary, haven’t fully played its role.

### Discussion of named entity model

Our NER model achieved 91.08% F-score without using any dictionary or externally annotated resource. The large classes such as “Body part” and “Medical discovery” generally perform well. The smaller classes perform less well as the data is not enough for the neural networks to learn good representations. For the rare classes, such as the “Medication” achieved the least performance because the medicine names are very diverse, a lot of unseen medicine names appeared in the test set. This is an indication that the only learn features from data maybe not enough yet as the data size is still small, and external medical knowledge is still required. Error analysis of some of the results also indicated that the word segmentation errors also propagated to the final classification, especially in medication. Some medicine names and laboratory tests are phonetic translation of English names or chemical molecular formulae, hence creates extra difficulty for the word segmentation.

## Conclusion

We presented the work of creating an annotated clinical corpus of Resident Admit Notes. The corpus is annotated with entities. We also present our preliminary experiments of named entity recognition using neural network approaches on our corpus. The model achieved better performance without using any feature engineering techniques and external resources. It is consistent with our high inter-annotator agreement in our annotation work. However, the entities in the corpus are not evenly distributed and the performance is bias to the major classes.

In the future, we will select sentences with rare entity types from admission summaries and try to balance the distribution of entities in our corpus, with the help of existing system. Currently our word embedding trained on non-medical documents, we will re-train the word embeddings on a larger collection of medical documents from the hospital to improve our system performance. And the deep research of the relationship between entities and modification work is also the next focus of our work.

## Abbreviations

CMER: Chinese electronic medical records
CNN: Convolution neural network
CRF: Conditional random fields
DA: discharge abstracts
EMRs: Electronic medical records
LSTM: Long short-term memory
NER: named entity recognition
NLP: Natural language rrocessing
RANs: Resident admit notes
